# Co-designing for behaviour change: The development of a theory-informed oral-care intervention for stroke survivors

**DOI:** 10.1080/24735132.2022.2096291

**Published:** 2022-07-21

**Authors:** Matthew Lievesley, Rachael Powell, Daniel Carey, Sharon Hulme, Lucy O’Malley, Wendy Westoby, Jess Zadik, Audrey Bowen, Paul Brocklehurst, Craig J. Smith

**Affiliations:** a School of Design, Northumbria University, Newcastle upon Tyne, UK; b Division of Psychology & Mental Health, School of Health Sciences, Manchester Academic Health Sciences Centre, University of Manchester, Manchester, UK; c Division of Cardiovascular Sciences, School of Medical Sciences, University of Manchester, Manchester, UK; d Manchester Centre for Clinical Neurosciences, Geoffrey Jefferson Brain Research Centre, Salford Royal NHS Foundation Trust, Salford, UK; e Division of Dentistry, School of Medical Sciences, Manchester Academic Health Sciences Centre, University of Manchester, Manchester, UK; f Stroke Survivor and Public Contributor in the research team, Leigh, UK; g Salford Royal NHS Foundation Trust, Salford, UK; h Geoffrey Jefferson Brain Research Centre, The Manchester Academic Health Science Centre, Northern Care Alliance & University of Manchester, Manchester, UK; i NWORTH Clinical Trials Unit, University of Bangor, Bangor, UK; j Division of Cardiovascular Sciences, School of Medical Sciences, Lydia Becker Institute of Immunology and Inflammation, Manchester Centre for Clinical Neurosciences, Manchester Academic Health Sciences Centre, University of Manchester, Manchester, UK

**Keywords:** Co-design, behaviour change, stroke care, oral-care, person-centred care, EBCD

## Abstract

This article discusses how research to understand the oral care needs and experiences of stroke survivors was translated into a prototypical intervention. It addresses the challenge of how to develop service improvements in healthcare settings that are both person-centred, through the use of co-design, and also based on theory and evidence. A sequence of co-design workshops with stroke survivors, family carers, and with health and social care professionals, ran in parallel with an analysis of behavioural factors. This determined key actions which could improve mouthcare for this community and identified opportunities to integrate recognized behaviour-change techniques into the intervention. In this way, behaviour change theory, evidence from qualitative research, and experience-based co-design were effectively combined. The intervention proposed is predominantly a patient-facing resource, intended to support stroke survivors and their carers with mouth care, as they transition from hospital care to living at home. This addresses a gap in existing provision, as other published oral-care protocols for stroke are clinician-facing and concerned primarily with acute care (in the first days after a stroke). Although it draws on the experiences of a single design project, this study articulates a ‘working relationship’ between design practice methods and the application of behaviour change theory.

## Introduction

The behaviour of individuals can have important impacts on health outcomes for patient populations, so to optimize health outcomes, interventions to support or change specific behaviours may be needed. To be effective, behaviour change interventions need to be acceptable and feasible to implement for relevant populations, and should also draw on appropriate evidence and theory (Craig et al. 2008). This article describes the methods and outputs of a multidisciplinary research team, developing an oral-health intervention. The development work engaged stroke survivors, their carers, and their clinicians in aiming to improve oral-health behaviours. The development process was guided by the Experience-Based Co-Design (EBCD) process (Robert 2013; Point of Care Foundation 2020), and the Behaviour Change Wheel (Michie, Van Stralen, and West 2011; Michie, Atkins, and West 2014).

The research team combined clinical specialists in stroke and oral health with experts in design and in health psychology, and points of conjunction and mutual learning across approaches are discussed. As such, this article presents a worked example of how design and health specialisms can collaborate to define a complex intervention. In particular, it addresses the challenge of how to develop interventions that are co-designed, to ensure acceptability to patients and health care professionals, and also based on theory and evidence. Such considerations are of relevance to designers and design researchers working in health or care settings, where design methods would benefit from a more explicit evidence base (Niedderer, Clune, and Ludden 2017).

### Case context

We aimed to develop an intervention to improve the oral health of stroke survivors living in the community by supporting self-care behaviours and enabling oral care support from carers. Each year, over 100,000 people in the UK have a stroke (Stroke Association 2018). Dental disease is highly prevalent in the stroke survivor population (Lyons et al. 2018, White et al. 2012). However, oral health is a relatively neglected part of stroke care (Horne et al. 2015; Talbot et al. 2005).

About a third of stroke survivors need help with activities of daily living after discharge from the hospital, and many experience disabilities that may impact their ability to manage their oral health (Stroke Association 2018), e.g. weakness in the hand/arm and swallowing problems. Research to date has largely focussed on oral care interventions for people hospitalized with a stroke (Lyons et al. 2018). How best to support stroke survivors with oral care after discharge into the community has received less attention, but it is important to ensure that appropriate support is in place for this population.

In Phase I of the research, we conducted interviews with 23 stroke survivors and focus groups with 19 health professionals to improve our understanding of the experience of stroke survivors and the context of the proposed intervention development (reported in O’Malley et al. 2020). Key issues identified included difficulties in carrying out oral hygiene self-care due to fatigue, forgetfulness and limb function, and dexterity problems. Routine seemed to be important for oral hygiene self-care but could be disrupted by hospitalization. For some, the aesthetic aspects of good oral care (e.g. a nice smile, fresh breath) were important. There appeared to be gaps in staff training and confidence in supporting patients with oral care, and problems with systems to ensure appropriate care was provided. Physical access to dental surgeries could be difficult.

### Research approach

In Phase II, reported here, we drew on findings from Phase 1 and used a co-design approach: Experience-Based Co-Design (EBCD) (Robert 2013; Point of Care Foundation 2020), alongside the Behaviour Change Wheel (Michie, Van Stralen, and West 2011; Michie, Atkins, and West 2014) to theoretically inform intervention development. Co-design treats service users as ‘experts by experience’ (Visser et al. 2005). Benefits can include improved fit between the service offer and users’ needs, better service experience, and higher satisfaction (Steen, Manschot, and Koning 2011). A co-design approach seeks to maximize both intervention acceptability to stakeholders and the feasibility of implementing an intervention. It can also benefit service providers by promoting collaboration between disciplines and growing organizational capacity for innovation (Steen, Manschot, and Koning 2011). EBCD was chosen as it enables a full range of healthcare service stakeholders to participate in patient-centred service improvement, is time-managed, and is a recognized model within the UK NHS, where it originated as ‘Experience-Based Design’ (Bate and Robert 2006; NHS Institute for Innovation and Improvement 2009; Robert 2013).

Whilst EBCD can be facilitated as a design process without expert designers, prominent early theories of co-design (Sanders and Stappers 2008) and recent empirical studies, suggest that using designer-facilitators has advantages in improving participants’ design capabilities and generating more innovative intervention outcomes (Dimopoulos-Bick et al. 2019; Ramos et al. 2020). In the current study, EBCD workshops were facilitated by experienced designer-researchers, drawing on a range of creative design tools to facilitate the co-design workshops, in line with the ‘designerly’ approach to co-design described by Robert and Macdonald (2017).

To make sense of the data emerging from the EBCD workshops, Service Design methods were chosen. Service Design methods involve the visualization of patient experiences on a timeline and are increasingly used to develop person-centred pathways in healthcare (Malmberg et al. 2019). We envisaged that articulating service-user-journeys for stroke survivors and their family carers, and integrating these into a Service Blueprint (Bitner et al. 2008) would provide a flexible way to visualize a joined-up provision despite the involvement of multiple provider organizations (Neilsen Norman Group 2020; Lievesley and Wassall 2015) and would guide the subsequent development of the intervention.

Although professional design agencies increasingly engage behavioural specialists (Lockton, Harrison, and Stanton 2010), most published case material on designing for behaviour change focuses on market impact without explanation of what principles, theories, or tools are used in development (Niedderer, Clune, and Ludden 2017). Behaviour change theories provide useful guidance when developing interventions by identifying the factors likely to be relevant to understanding, predicting, and changing behaviour. The Medical Research Council (MRC) guidance for developing and evaluating complex interventions highlights the importance of drawing on existing evidence, and of either identifying or developing a theory on which to base an intervention (Craig et al. 2008).

There are many decisions to be made when designing an intervention, for example: which theoretical factors are most relevant and most feasible to target in a specific context? The Behaviour Change Wheel is a framework designed to guide the researcher in negotiating such issues (Michie, Atkins, and West 2014: Michie, Van Stralen, and West 2011). At the centre of the ‘Wheel’ is the COM-B model, which proposes that ‘Capability’, ‘Opportunity’, and ‘Motivation’ interact in producing ‘Behaviour’. Capability is ‘the individual’s psychological and physical capacity to engage in the activity concerned’ (includes knowledge and skills); opportunity is ‘the factors that lie outside the individual that make the behaviour possible or prompt it’; motivation is ‘all those brain processes that energize and direct behaviour’ (includes conscious processes as well as habitual and emotional processes) (Michie, Van Stralen, and West 2011, 6). The next layer of the Wheel is ‘intervention functions’ (ways in which an intervention can change behaviour): education, persuasion, incentivization, coercion, training, enablement, environmental restructuring, and restrictions. In the outer ring are policy categories that would support intervention delivery, e.g. guidelines, legislation, service provision, and fiscal measures.

A related approach is the Theoretical Domains Framework (TDF), which synthesized theoretical factors from a range of theories, using a consensus approach, yielding 14 theoretical domains (Cane, O’Connor, and Michie 2012). The TDF domains relate to the broader COM-B categories and can be used in conjunction with the Behaviour Change Wheel, supporting a more detailed behavioural analysis (Michie, Atkins, and West 2014).

In summary, this study aimed to produce an intervention with maximal acceptability and fit with existing patterns of care, and optimal potential for enhancing behaviours relevant to stroke survivor oral health by combining a co-designing approach (EBCD), with behaviour change theory (Behaviour Change Wheel and TDF).

## Methods

### Overview

The research was conducted in the North West of England and explored the experiences of stroke survivors treated in UK National Health Service (NHS) hospitals. Health and social care professionals (HSCPs) were also based within publicly funded services or relevant regional, voluntary sector organizations. The study was reviewed and granted favourable ethical opinion by the NRES Committee Northwest Haydock Research Ethics committee (REC Ref No: 17/NW/0335). The study was conducted according to the standards of the European Medicines Agency Guidelines for Good Clinical Practice (Kingham, Bogaert, and Eddy 1994).

**Figure 1.**
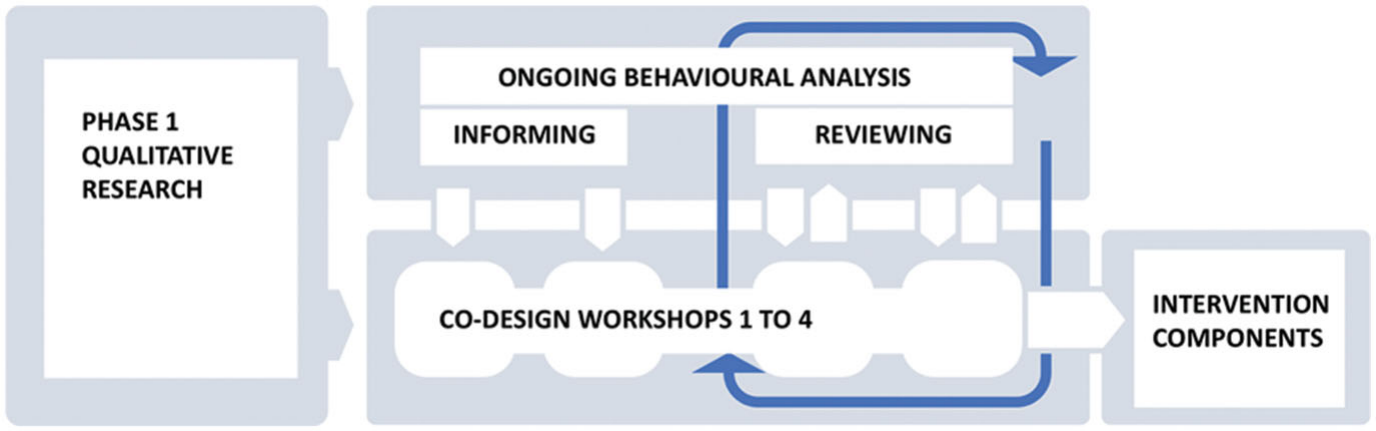
The behavioural analysis and the co-design workshops worked in parallel throughout Phase 2, to develop the Intervention Components.

A behavioural analysis, informed by the Behaviour Change Wheel staged approach, was combined with four co-design workshops across 9-weeks, involving either stroke survivors (with family carers), HSCPs, or both. We describe the use of the Behaviour Change Wheel first, and then the experience-based co-design workshops. In reality, the two processes were iterative rather than chronologically separate (see Figure 1).

### Use of the behaviour change wheel to inform intervention development

Stages outlined in Michie, Atkins, and West (2014) guide to using the Behaviour Change Wheel were followed.

#### Understanding the behaviour

This stage aimed to: specify what the behavioural problem is; specify the behaviours that need to change and who needs to carry out the behaviours; and understand what the behavioural determinants are (the factors are that need to change to change the specified behaviour). This stage was led by a Health Psychologist with expertise in behaviour change (author Powell), in parallel with the Phase 1 thematic analysis of qualitative data (by author O'Malley), such that the understanding of the behaviour was grounded in the Phase 1 data.

The target oral hygiene behaviour was selected as cleaning the teeth/mouth twice a day, a behaviour that would be performed by the stroke survivor, an informal caregiver, or professional caregiver. The target dental care behaviour was: accessing dental care (e.g. visiting dentist), a behaviour performed by the stroke survivor. Detailed initial specifications of these two behaviours are provided in Tables 1a and 1b.

**Table 1a.** Initial specification for target behaviour ‘cleaning teeth/mouth’.

Target behaviour: ‘cleaning teeth/mouth’	
Who needs to perform it	Stroke survivor OR informal caregiver OR professional caregiver
What does the person need to do differently?	Increase cleaning frequency
	Improve cleaning skill
When will they do it?	Morning, evening
Where will they do it?	In home of stroke survivor. Bathroom for mobile stroke survivors; may be managed in another room if less mobile.
How often will they do it?	Twice/day
With whom will they do it?	Either by self, or in collaboration with caregiver (caregiver would carry out behaviour with the survivor).

**Table 1b.** Initial specification for target behaviour ‘accessing dental care’.

Target behaviour: ‘accessing dental care’	
Who needs to perform it	Stroke survivor
What does the person need to do differently?	Attend regular dental appointments
When will they do it?	6 monthly/yearly, or when have a problem
Where will they do it?	Dental clinic
How often will they do it?	6 monthly/yearly, or when have a problem
With whom will they do it?	May manage with the support of a caregiver (informal or formal).

We used the Theoretical Domains Framework (TDF) to guide our identification of behavioural determinants. For example, one domain within this framework is ‘physical skills’. The TDF, therefore, guides us to consider whether ‘physical skills’ is a factor affecting whether or not someone carries out oral care behaviours, and Phase 1 data would enable an understanding of how physical skills might be important in the present context (e.g. impaired hand functioning affecting the ability to clean teeth).

The identification of behavioural determinants was initially conducted based on the analysis of Phase 1 data. This behavioural analysis was further informed by discussions from the first two EBCD workshops. Supplementary Appendix 1 contains details of this process for the behaviour of cleaning teeth/mouth by stroke survivors as an example. Important factors influencing this specific behaviour appeared to be: physical skills (e.g. strategies to clean teeth despite physical limitations); knowledge; cognitive skills; memory (forgetting was common, especially when tired); behavioural regulation (importance of habit/routine); environmental context (e.g. tools; access to bathrooms); social influence (e.g. practical help and reminders from caregivers); beliefs about consequences; goals; emotion.

For the behaviour of cleaning teeth/mouth by caregivers, important factors seemed to be: physical skills; knowledge; cognitive and interpersonal skills; environmental context and resources (e.g. time; the need for oral care to be specified in care plans); social influences; professional/social role and identity; beliefs about capability (lack of confidence in cleaning another’s teeth seemed common); and beliefs about consequences and emotion.

Finally, important factors determining accessing dental care by stroke survivors appeared to be: knowledge (including knowing how to find a suitable dentist); cognitive and interpersonal skills; memory; environmental context, and resources (e.g. availability of NHS dentists willing to accept stroke survivors; accessibility of practices); and social influences.

This behavioural analysis informed the EBCD process, ensuring that issues identified as potentially important during Phase 1 and initial workshops were considered as intervention development progressed.

#### Identifying intervention options and potential content

Potential intervention functions and policy categories were identified following guidance by Michie, Atkins, and West (2014). For example, ‘training’ was identified as an intervention function to support the need for physical skills, and potential policy categories for the intervention function ‘training’ included service provision and guidelines. Possible behaviour change techniques were then identified. A behaviour change technique is ‘an active component of an intervention designed to change behaviour’ (Michie, Atkins, and West 2014, 145). A taxonomy of behaviour change techniques has been developed to optimize accurate reporting and understanding of intervention components (Michie et al. 2013). For example, one behaviour change technique is prompts/cues (‘Introduce or define environmental or social stimulus with the purpose of prompting or cueing the behaviour’, Michie, Atkins, and West 2014, 268). Author Powell mapped potential intervention functions to possible behaviour change techniques (see Supplementary Appendix 2 for an example of this process for the behaviour of cleaning teeth/mouth by stroke survivors).

**Figure 2.**
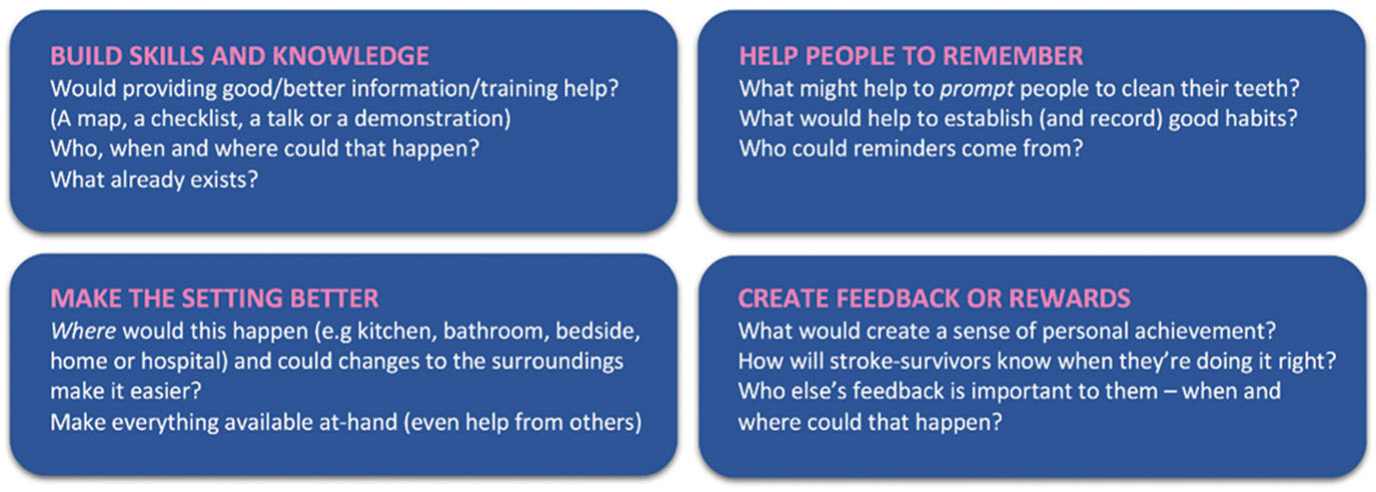
A prompt document with four sections, drawing on approaches from the behavioural analysis, was used to encourage discussion and generate improvement ideas.

This theoretical-based approach does not dictate the exact content of an intervention but instead provides a range of options for consideration. In the present study, this staged analysis informed discussions in the early EBCD workshops. For example, it was used as the basis for four plain-language, flash-card-style prompts to stimulate ideas from Workshop participants (see Figure 2). The prompts indicated broad approaches within which specific ideas could be defined. In this way, evidence and theory were used to inform and scaffold the co-design process, but did not dictate intervention design. For the intervention to be truly responsive to the needs and desires of stroke survivors and professionals, it was important to prioritize their views as to how the challenges of the target behaviours could be addressed—we did not wish to restrict discussions or the creativity of the EBCD process.

During EBCD workshops and through interactions with the research team led by the first author, speculative intervention components were suggested, which addressed the behavioural analysis outlined in Stage 1 above. Author Powell analysed these, identifying which behaviour change techniques were being used in each case. They also worked with the design authors to advise on issues, such as wording, where the designer’s intent was in line with a specific behaviour change technique, but minor changes were needed to maximize the chances of effectively promoting behaviour change. This approach would ensure that components would contain identifiable ingredients that would be theoretically expected to be effective. This mapping was regularly updated and developed as the intervention became more clearly defined through the design process. The final version is shown in Table 2.

### Use of experience-based co-design to develop the intervention

Four EBCD workshops enabled service users and staff to explore key issues and to define the key elements and format of the proposed intervention, with the aim of understanding and increasing the acceptability of the end result (Diepeveen et al. 2013).

#### Co-design participants

Seven stroke survivors, two family carers, and 16 HSCPs took part in at least one of four co-design workshops between March and May 2019. Stroke survivors’ ages ranged from 41 to 70, four were male, and three were female. Five had experienced a single stroke; two had experienced two strokes. The time since the most recent stroke ranged from one to eight years. Both carers were female and aged in their sixties. The HSCP participants included individuals with roles in hospitals, community healthcare, social care, and voluntary sector services, representing all stages in the sequence of stroke care. They were dental/oral health practitioners (including dental nurses) (6), occupational therapists (2), stroke nurses (2), speech and language therapists (2), stroke support officer (1), social worker (1), dietician (1), health care assistant (1). Years since qualification ranged from 4 to 38, and the number of years’ experience working with stroke survivors ranged from 3 to 34.

#### Procedure

The aims of the EBCD process were to consider the target behaviours, behaviour change techniques, and the content and mode of delivery of the proposed intervention. Workshop activities also explored barriers to implementation and optimal timing for the provision of the intervention. All workshops included a briefing, making the EBCD process transparent to all participants (following Reay et al. 2017), and up to 90 min of activities. They were conducted within a hospital or community-centre settings. Data were collected as audio recordings, facilitators’ notes, paper mock-ups of possible intervention components (prototypes), and sticky notes written by participants, assembled into visual documents. These documents and co-created prototypes were the primary data outputs from the workshops, with audio recordings used to support the recall.

*Trigger-films* (Point of Care Foundation 2020) were used to punctuate each of the co-design workshops, setting the agenda, along with a series of paper-based activities, prompts, and workbooks. Constructed using video clips from Phase I interviews, the films reinforced the patient voice within the design process (Point of Care Foundation 2020; Ramos et al. 2020) and brought focus to key topics identified in the behavioural analysis, creating a candid portrait of current service provision.

**Workshop 1**: Stroke survivors and carers explored whose advice they had found important during their recovery and how they would advise others entering the care of a stroke service. Different stroke-survivor Personas (Jones 2013) were developed for this purpose. The participants were encouraged to share observations about their lived experiences of care at all stages of the stroke service journey.

**Figure 3.**
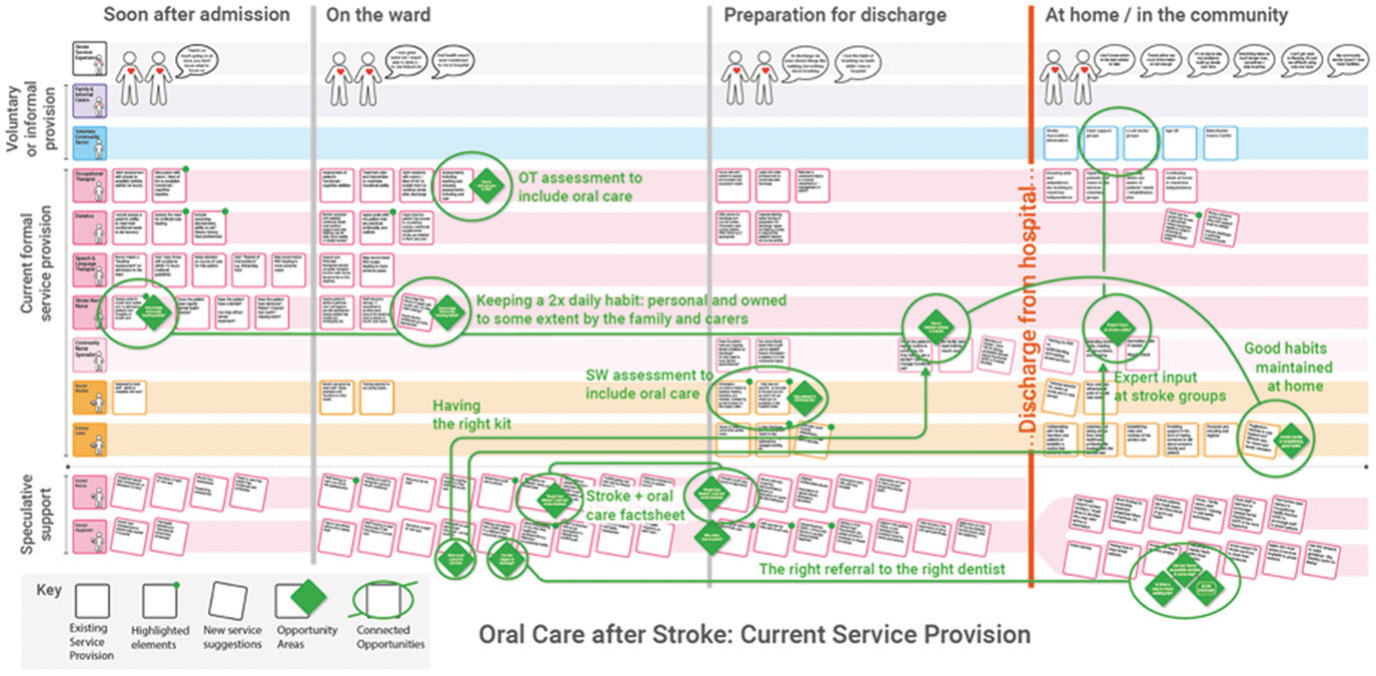
Map of existing service-provision at the research site, discipline by discipline, across 4 time phases: *Soon after admission (hospital) | on the ward | preparing for discharge | at home/in the community.*

**Workshop 2**: The HSCPs constructed a step-by-step map of current service provision, covering pre- and post-discharge (Figure 3). Each participant’s activities and contributions to care were noted and organized into individual sequences, before being aggregated into the mapping document, which stimulated a rich discussion. Seven disciplinary specialisms were represented, highlighting key steps, and the temporal relationships between them, which comprised the current provision.

Participants were asked to respond to a range of written challenges e.g. ‘some stroke survivors lose their brushing/care habits during their hospital stay’. The HSCPs identified changes that might be made to address these challenges. To support the discussion, four prompts were used (Figure 2). The four areas were consistent with intervention functions identified as being potentially relevant in the earlier behavioural analysis: education, persuasion, training, environmental restructuring, modelling, enablement, and incentivization.

**Design Synthesis 1:** Design Synthesis 1 involved reflecting on data generated in workshops 1 and 2, and the Phase 1 qualitative findings, in relation to the target behaviours. The synthesis involved the physical organization and reorganization of the data in search of relationships or patterns that could be translated into actionable insights (English 2006; Kolko 2010). In this study, the stroke-survivor and HSCP workshop data were reorganized along a common timeline, highlighting potential *opportunity* *areas* identified by both sets of stakeholders. These were phrased as successful outcomes, for example: ‘*good habits maintained at home’* or *‘having the right kit’*, and were then overlaid onto the map of existing service provision built during Workshop 2. The digitized version of the map created in Workshop 2 is shown here, complete with all opportunity areas identified overlaid as green diamonds. Green lines connect recurring themes.

The opportunity areas were sequenced into an improved User Journey. Following the Service Blueprinting method (Bitner et al. 2008), the details of this User Journey were explored, in terms of the people, places, artefacts, and responsibilities needed to realize improvements (Neilsen Norman Group 2020). The improvement ideas were developed into a set of prototypical intervention components to develop in Workshops 3 and 4. They were mainly in the form of printed paper mock-ups, unfinished, but coherent enough to provoke discussion, critique, acceptance, or rejection (Wensveen and Matthews 2015).

**Workshop 3:** Stroke survivors and carers reviewed, critiqued, and improved the prototypes, in terms of information content, legibility of text and images, and timing/modes of use. Feedback was recorded by the facilitators, and recommended adaptations were made to the mock-ups. These refined prototype proposals were also checked against the behavioural analysis. This step was important to ensure that key behavioural determinants of the target behaviours were being addressed and to identify where behaviour change techniques might be clearly relevant but absent.

**Workshop 4:** Stroke survivors, carers, and HSCPs together, examined each prototypical intervention component through two activities: First, each participant reviewed 2 or 3 components, selected according to their own expertise. In personalized workbooks, they answered questions, such as: *How would this best fit into existing patterns of workflow? (who? where? when?)*; and: *What would you add to this idea?* Next, the cohort split into three themed groups to discuss the means and viability of implementing the proposed components. Together, these activities provided a structured critique of the components.

**Design Synthesis 2:** Data collected in all workbooks, and all adaptations or suggestions made directly to the mock-ups, were transcribed to a spreadsheet for cross-referencing and used to inform the final round of prototype development. Where specific additional expertise was needed, e.g. to agree on terminology, or to understand commissioning processes, the research team sought that advice after the workshops.

Based on this final input from all stakeholders, the intervention components were rationalized in number and final design refinements were made. They were organized into three subsets based on the timing of their provision and the draft *Service Blueprint* was updated. Together, this set of mock-ups and the corresponding Blueprint define the proposed intervention.

## Results

In this section, we describe the Improved User Journey, the Service Blueprint, and a short summary of the 13 potential intervention components.

### Improved user journey

**Figure 4.**
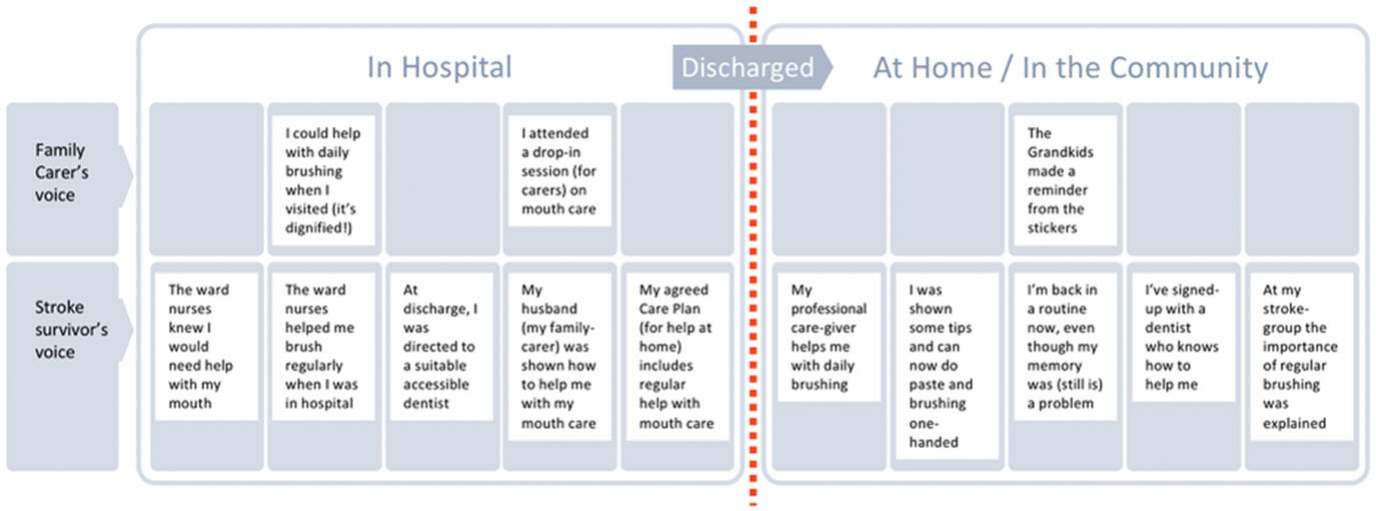
The improved user journey proposed—expressed in the voices of an example stroke survivor and their family carer.

The Improved User Journey is composed of the series of encounters that a stroke survivor and their informal carer might experience in a revised and improved service (see Figure 4). The following examples illustrate how the data from the workshops were cross-matched to identify where suggestions from the different participant groups related closely to each other, and how this synthesis process informed the improved user journey.

*Example 1: engaging family carers:* In Workshop 2, a dental professional talked about having an ‘oral health kit with appropriate resources – not the usual kit’ for in-hospital mouthcare – and a Stroke nurse suggested ‘encourage the family to assist with mouth care—Do they need training?’. Thinking about people returning home, a community-based nurse asked ‘do family carers need training in mouthcare?’ and the Social Worker identified an opportunity to provide ‘training on oral hygiene and denture care – for carers and family members’. This resonated with Workshop 1 data from stroke survivors ‘being given the wrong things at the wrong time’ and also with a finding from the Phase I interview data – that the stroke and consequent hospital stay can interrupt a person’s pre-existing good-habits – leading to a loss of daily brushing (O’Malley et al. 2020).

*Example 2: mouthcare in the care plan:* An Occupational Therapist (OT) had highlighted that not all OTs checked whether stroke survivors due to be discharged could brush their own teeth – because it is not currently on the EPR (the Electronic Patient Record), which serves as a discharge checklist. Similar EPR limitations were raised by a Social Worker regarding their Care Needs Assessment tool, which determines the support a person leaving hospital will receive from professional caregivers, once they return home. If mouthcare is not included as an EPR check-box at discharge, it could be overlooked, resulting in professional caregivers not being instructed to provide it post-discharge.

The series of opportunity areas synthesized from the Workshop data in this way were first mapped to current service provision (Figure 2) and then refined and organized into a notional, improved user journey (Figure 4). This was formulated in the voices of a stroke survivor and carer to explain an improved experience of care in a person-centred way. For example, Family Carer’s voice: ‘I attended a drop-in session on mouth care’; Stroke survivor’s voice: ‘My husband (family-carer) was shown how to help me with my mouth care’.

### Service blueprint

**Figure 5.**
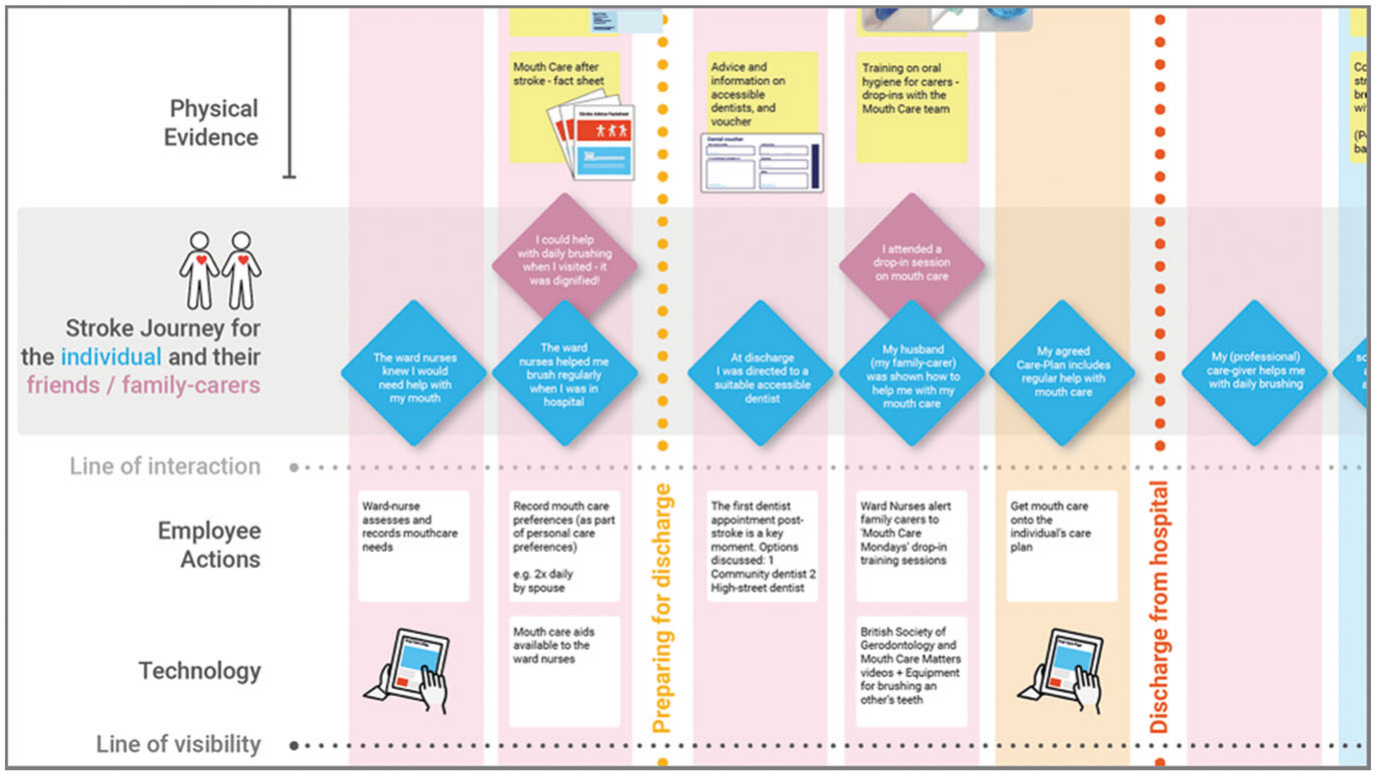
Excerpt from the Service Blueprint drafted to synthesize the findings from Workshops 1 and 2 and to define a range of prototypical elements of the intervention. The full document can be viewed in Supplementary Appendix 3.

The Improved User Journey was then used as the core of a Service Blueprint, based on Neilsen Norman’s format (2020), an excerpt of which is shown in Figure 5.

A range of potential intervention components was identified in relation to the user journey. When these were cross-checked to the behavioural analysis, areas which could be enhanced, such that intervention components aligned with recognized behaviour change techniques, were identified.

*Example 3: learning new techniques:* It was identified that when helping individuals to learn how to carry out behaviours, the behaviour change technique ‘Demonstration of the behaviour’ was relevant (i.e. ‘showing’ could be beneficial in addition to ‘telling’). This tied into an insight from Workshop 3, that smartphones were regularly used on the ward by family visitors, usually for looking-up medical or pharmaceutical information. With these insights, a proposed *factsheet* (C2 below) was amended to incorporate links to web-based video demonstrations (such as how to safely brush another person’s teeth).

Other rows in the Blueprint enabled the research team to consider supporting actions and resources needed to make each step possible. It also enabled the likely ownership of each element to be considered across the range of health, social care, and voluntary sector organizations that may be involved in the delivery of the intervention. In all, the Blueprinting process identified thirteen potential intervention components, which could support the improved user journey.

### Potential intervention components

The potential intervention components developed are summarized in Table 2. Alongside, are the Behaviour Change Techniques included in the final versions of each. An expanded table including Intervention Functions and Policy Categories is available in Supplementary Appendix 4.

**Table 2.** The set of prototypical intervention components is identified through the synthesis process.

	Intervention component and modality	Behaviour change techniques (BCTs)
C1	POSTER PROMPT for the Ward bathroom	Target: stroke survivors 7.1: Prompts/cues
C2	FACTSHEET Mouth care after Stroke. Given on admission to the stroke ward, at Mouth Care Mondays (C5) and at Stroke Groups (C13)	Target: stroke survivors and family carers 4.1 Instruction on how to perform the behaviour 5.1 Information about health consequences, 5.3 information about social/environmental consequences; 6.1 Demonstration of behaviour [if follow QR link] [2.2—feedback on behaviour—advised on how to gain feedback]
C3	WEEK 1 MOUTH CARE CHART Bedside chart. Tear-off mini brushing-packs for the first week on the stroke ward.	Target: stroke survivors and family carers 2.3 Self-monitoring of behaviour 7.1 Prompts/ cues 12.5 Adding objects to environment;
C4	WEEK 2 MOUTH CARE CHART Bedside chart.	Target: stroke survivors and family carers 2.3 Self-monitoring of behaviour 7.1 Prompts/ cues 12.5 Adding objects to environment;
C5	MOUTH CARE MONDAYS Carers’ weekly drop-in—on the ward. One-hour draft agenda (and poster C6)	Target: family carers 3.1 Social support (unspecified) 4.1 Instruction on how to perform the behaviour; 5.1 Information about health consequences, 5.3 Information about social/environmental consequences; 6.1 Demonstration of the behaviour 8.1 Behavioural practice [if feasible] 12.5 Adding objects to the environment [if feasible to provide useful equipment]
C6	MOUTH CARE MONDAYS Poster for the ward’s notice-board and/or Family Room	Target: family carers. Target behaviour: attending Mouth Care Mondays. 4.1 Instruction on how to perform the behaviour 7.1 Prompts/cues
C7	EPR CHANGE 1 (software fix) For action by Occupational Therapists	Target: Occupational Therapist Target behaviour: check for independent oral self-care 2.3 Self-monitoring of behaviour 7.1 Prompts/cues
C8	DENTAL VOUCHER Issued at discharge, it secures a *double* dental appointment for the named recipient, ensuring they aren’t rushed.	Target: stroke survivors. 7.1 Prompts/cues Target: dental practices. 10.1 Material incentive (behaviour) 10.2 Material reward (behaviour)
C9	EPR CHANGE 2 (software fix) For action by Hospital-based Social Workers	Target: Social Worker. Target behaviour: check for independent oral self-care 2.3 Self-monitoring of behaviour 7.1 Prompts/cues
C10	TRAINING ON MOUTH CARE—FOR PROFESSIONAL CAREGIVERS	Target: professional caregivers. Likely to include: 4.1 Instruction on how to perform the behaviour; 5.1 Information about health consequences, 5.3 information about social/environmental consequences; 6.1 Demonstration of the behaviour 8.1 Behavioural practice
C11	PEER-TO-PEER FILMS Hopeful online messages, shared by stroke-survivors	Target: stroke survivors Likely BCTs (depending on final content of films): 9.1 Credible source 15.1 Verbal persuasion about capability 16.3 Vicarious consequences 6.1 Demonstration of the behaviour
C12	MEMORY STICKERS For the home environment—given at discharge	Target: stroke survivors 7.1—Prompts/cues
C13	EXPERT INPUT AT STROKE GROUPS Once or twice a year	Targets: stroke survivors and family carers 3.1 Social support (unspecified) 4.1 Instruction on how to perform the behaviour 5.1 Information about health consequences, 5.3 Information about social/environmental consequences; 6.1 Demonstration of the behaviour 8.1 Behavioural practice [if feasible] 12.5 Adding objects to the environment [if feasible to provide useful equipment]

*Note*. The target behaviours are usually those originally specified: ‘cleaning teeth/mouth’ and ‘accessing dental care’. Additional, intermediate target behaviours (identified during the research) are indicated where relevant. BCTs are numbered according to Behaviour Change Technique Taxonomy (v1) (Michie et al. 2013).

### Prioritized intervention components

**Figure 6.**
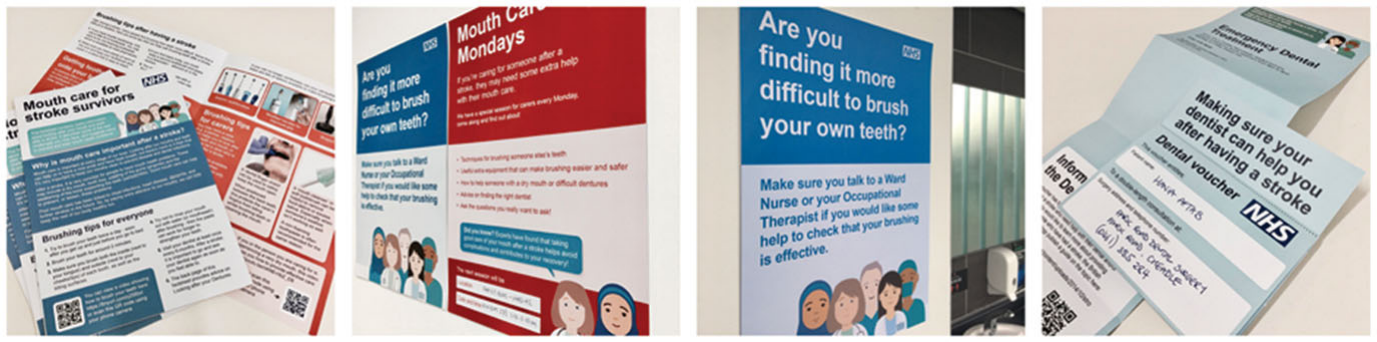
Examples of the co-designed components of the intervention.

The feedback from Workshop 4 participants, on how the proposals might best fit into existing patterns of care, helped to prioritize (see Supplementary Appendix 4) and cluster nine of the original thirteen intervention components around three milestones in care, i.e. key opportunities for intervention within the stroke-journey (see examples in Figure 6).

**Table 3.** Proposed timing of delivery of the prioritized intervention components.

	When	What
1	On admission to the ward	Mouth Care Charts: week 1 with cleaning packs (C3) and week 2 without (C4) Factsheet (C2)—Mouth care for stroke survivors and carers Mouth Care Mondays (C5 + C6)—Carers’ weekly drop-in training session: defined through an outline agenda, its frequency, and a promotional poster.
2	At the point of discharge	Poster Prompt (C1): prompts a check that the person can brush independently Dental Voucher (C8): guides stroke survivors to a double appointment with a good dentist Memory Stickers (C12) bathroom-mirror reminder-stickers: to apply at home with a family carer(s)
3	At home/in the community	Expert input at Stroke Groups (C13)—Mouth Care Q + A and equipment demos at local Stroke Groups /lunch clubs. Agenda based on (C5) Mouth Care Mondays and sessions led by either Dental Therapists or SLTs. FACTSHEET (C2) was given to participants.

## Discussion

In this study, behaviour change theory, evidence from qualitative research, and experience-based co-design were effectively combined to develop the structure, format, and content of an intervention promoting good mouth care for stroke survivors. The proposed intervention aims to enhance the oral-care support needed by stroke survivors following hospital discharge, where appropriate provision has previously been poorly defined (Lyons et al. 2018). It includes guidance on self-care, accessing a suitable dentist; getting help from professional care-givers; managing memory problems; and support provided via community-based groups.

The intervention as a whole is in prototypical form, with components that are developed to different levels of readiness for implementation. For example, the Factsheet (C2), Mouth Care Mondays agenda and poster (C5 + C6), and Expert Input at Stroke Groups (C13) are complete and fully defined. Other elements, such as the Toothbrushing Charts (C3 + C4), are well-defined in terms of what they are and why they are needed, but the complex set of interactions they invoke, between families and staff and the ward environment, needs further testing and iteration. This will be the subject of follow-on work.

Design methods, particularly in co-design and particularly in complex settings, such as healthcare, put emphasis on making progress and improvement rather than pursuing perfect, complete solutions (Norman and Stappers 2015). The lead workshop facilitator’s previous healthcare design experience was an important enabler (following Ramos et al. 2020) in encouraging and exploring ways to overcome blockers. In our study, for example, two desirable changes to the Electronic Patient Records (EPR) system, identified in Workshop 2 (C7 + C9), would have been optimal ‘solutions’ by mandating certain actions but were confounded by a long (2-year) delay between software-change-cycles. These two technology-driven checks were successfully ‘reframed’ (Dorst 2011) as *conversations we want to happen*, and the following person-centred alternatives developed instead. First, the Poster Prompt for the Ward Bathroom (C1) promotes the idea of independent brushing to all and explicitly grants permission to patients to ask about it. Second, the Voucher (C8) prompts a specific conversation with the Social Worker at the point of discharge, drawing attention to a mouth care needs-assessment—substituting for an EPR-mandated check (C9). So, whilst the EPR changes remain a longer-term goal, in the interim, these two alternatives still prompt relevant and timely actions in the care pathway. The alternatives described demonstrate the capacity of design to promote flexible and pragmatic approaches, to confront institutional barriers, and still make progress (Cross 2011).

A theory-informed and evidence-based development process was followed and the theoretical tools and frameworks used are made explicit, answering Niedderer’s call for better-documented processes from design teams (Niedderer, Clune, and Ludden 2017), and MRC recommendations for complex intervention design (Craig et al. 2008). The design practice community will recognize the importance of being able to demonstrate an explicit and consistent approach to defining behaviour as an integrated part of design methods.

At first glance, the two approaches combined in this study may appear to be very different. The lead author is a Designer-Researcher and led the EBCD approach, which centres on design group participants’ experiences and expressed needs, and creative development of an intervention. The second author is a Health Psychologist who led the use of the Behaviour Change Wheel approach, which proposes a pathway to intervention design in line with available evidence and theoretical frameworks. However, co-design workshops produce a type of evidence that can feed into the Behaviour Change Wheel approach. Further, the Behaviour Change Wheel approach proposes that interventions are assessed against APEASE criteria: affordability; practicability; effectiveness and cost-effectiveness; acceptability; side-effects/safety; and equity (Michie, Atkins, and West 2014), issues that are likely to be considered in an EBCD group.

Nevertheless, the differences in approach between these two researchers needed some consideration. Whilst both disciplines value the experiences, observations, and insights of the EBCD group members, the Health Psychologist’s approach also focussed on incorporating previous evidence (from the literature and from the Phase 1 related research) and behaviour change theory into intervention design. This study was the first time that these two researchers had worked together to integrate the two approaches, and there were, at times, differences in language and approach that neither had anticipated. Working through these matters led to beneficial learning for both parties, a research process, and the final result, that neither discipline could have achieved alone. We would recommend that researchers from two such different disciplines build in time to: articulate their expectations and assumptions early in the process; to jointly shape the EBCD workshop agendas and tools; and to reflect together on the data generated after each workshop.

Useful intersection points have been identified through this case, where behavioural theory can augment design methods without restricting the creative process. First, by using the Behaviour Change Wheel (BCW), target behaviours in both the stroke survivor and family/informal carer populations were made explicit (Table 1), strengthening and clarifying the design brief. Second, drawing on evidence gained in phase 1 interviews, the use of the BCW allowed the identification of potential intervention functions and behaviour change techniques. These findings were used as prompts (Figure 2) to scaffold (not prescribe) the co-designing workshop activity. Third, in the synthesis work between workshops, coding each intervention component using the Behaviour Change Technique taxonomy (Michie et al. 2013) helped to tailor the intervention content to maximize effectiveness (detailed in Tables 2 and 3). This also means that each intervention component is clearly defined in terms of its behaviour change ingredients, which enables the accurate reporting of the intervention components and will facilitate replication and future evaluation.

Other health intervention development research has combined behaviour change theory with co-design interventions, but the process for combining approaches has varied. Some used behaviour change models, such as COM-B to construct prototypical intervention components, before co-design workshops (e.g. Aljaroodi et al. 2017; Bonner et al. 2019). At least one study has successfully engaged patients as co-designers during the later stages of a theory-informed intervention development process (e.g. Salmon et al. 2019). In our research, we drew on co-design input both to explore initial priorities and also to shape two cycles of prototype development and integrated behaviour change theory throughout, i.e. to inform each workshop agenda and to evaluate and optimize emerging intervention prototypes.

The intervention development described was based on a single locality, so it will be important to gain wider feedback on the intervention components, to ascertain their fit with practice across other regions. The next stage will involve evaluating the effectiveness of the intervention in improving oral health care in stroke survivors.

## Conclusions

This project has addressed a highly complex health issue, combining methods from Design and Health Psychology to develop an intervention aiming to maximize acceptability, the feasibility of implementation, and effectiveness. The outcome is predominantly a patient-facing resource intended to provide continued support for stroke survivors post-hospital discharge, and as such will address a gap in current provision.

Design, with its user-centred philosophy, has great potential to help health and care service providers make progress towards genuinely person-centred care. However, the design’s socially constructed approach does not easily demonstrate the rigour valued by healthcare commissioners. Through this case, a theory-informed development process has been described where the rigour is made explicit as part of the project outputs. It articulates a ‘working relationship’ between design practice methods and the application of behaviour change theory.

## Supplementary Material

Supplemental Material
