# Supplementary material for: Co-designing for behaviour change: The development of a theory-informed oral-care intervention for stroke survivors
Source: Design Health (Abingdon). 2022 Jul 21;6(2):221–43. doi: 10.1080/24735132.2022.2096291 (PMC9612934; doi:10.1080/24735132.2022.2096291)
Supplement: Supplemental Material [file RFDH_A_2096291_SM0665.docx]

**Appendices** - Co-designing for behaviour change – The development of a theory-informed oral-care intervention for stroke survivors.

Corresponding author: matthew.lievesley@northumbria.ac.uk

Figshare link to this set of appendices:

DOI: 10.25398/rd.northumbria.14954913

**APPENDIX 1**

Table mapping the identification of behavioural determinants for the behaviour of cleaning teeth/mouth by stroke survivors, structured using the COM-B model (Capability, Opportunity, Motivation, Behaviour) and the TDF (Theoretical Domains Framework).

| **COM-B Component** | **TDF domain** | **What are issues for stroke survivors? (from Phase 1 findings and Phase 2 workshops)** |
| --- | --- | --- |
| Capability: physical | Physical skills | Important where physical impairments are present – e.g. only 1 hand; if needing to clean with non-dominant hand – need to learn physical techniques/strategies to manage despite limitation.  Need to know how to use new materials/equipment  Vision difficulties: may be difficult to see white paste |
| Capability: psychological | Knowledge | Lack of knowledge identified regarding reasons for oral hygiene and the recommended behaviours (what should do, how often; use of mouthwash, rinsing, brushing, floss; how to clean dentures).  Lack of knowledge around wider health complications resulting from poor oral hygiene. |
|  | Cognitive and interpersonal skills | Need to recognise that need to clean teeth and have the cognitive skills to carry it out. |
|  | Memory, attention and decision processes | Forgetting common (especially when tired); prompts to clean helpful. |
|  | Behavioural regulation | Development and maintenance of habit /structured routine important. Hospitalisation can disrupt habit – but also opportunity to develop habit where none pre-stroke? |
|  | | |
| Opportunity: physical | Environmental context and resources | Tools e.g. flip top, pump-action toothpaste; electric toothbrush/tools to facilitate use of toothbrush; alternatives to dental floss; coloured paste (vision problems); mirror for feedback.  Access to bathroom where mobility limited.  Need to be able to reach equipment.  Priorities: limited time/energy resources; a lot to manage; not necessarily seen as high importance. |
| Opportunity: social | Social influences | Importance of family/caregivers: reminding to clean teeth; providing advice as to what should do; helping with tasks/equipment/cleaning where physical limitations.  Communication challenges: difficult to ask someone to help/to clean (either due to speech problems –or feels awkward/embarrassing. |
|  | | |
| Motivation: reflective | Beliefs about consequences | Often perceived as important; some focussed on importance regarding aesthetic consequences of cleaning/not cleaning, rather than health consequences. High value of aesthetic aspects indicated importance of being able to, and having confidence to, interact socially.  For some: lower priority, particularly initially post-stroke; not seen as central to health.  Feels unpleasant if not clean  Maintain independence.  Someone else cleaning teeth: unpleasant; lack of dignity |
|  | Goals | Avoid teeth falling out/false teeth; avoid rotting gums; nice-looking teeth; avoid smelly breath; feel better; avoid costs of dental treatment |
| Motivation: automatic | Emotion | Mention of being ‘lazy’ despite wanting to clean teeth. Reflects fatigue and/or depression?  Might have changed emotional states (e.g. feeling angry)  Important to regain self-esteem and confidence post-stroke – then take more interest in self-care (showering, cleaning teeth).  Guilt/frustration in asking for help.  Overwhelmed initially; can be over-optimistic re what will be able to achieve. |
|  | | |

**APPENDIX 2**

Mapping of behavioural determinants to potential intervention functions, policy categories and behaviour change techniques for the behaviour of cleaning teeth/mouth by stroke survivors.

| **TDF domains** | **Intervention functions** |
| --- | --- |
| Physical skills | Training |
| Knowledge | Education |
| Cognitive and interpersonal skills | Training |
| Memory, attention and decision processes | Training, environmental restructuring, enablement |
| Behavioural regulation | Education, training, modelling, enablement |
| Environmental context and resources | Training, restriction, environmental restructuring, enablement |
| Social influences | Restriction, environmental restructuring, modelling, enablement |
| Beliefs about consequences | Education, persuasion, modelling |
| Goals | Education, persuasion, incentivisation, coercion, modelling |
| Emotion | Persuasion, incentivisation, coercion, modelling, enablement |
|  | |

***Table App2a:*** *Mapping relevant TDF domains to potential intervention functions for the behaviour of cleaning teeth/mouth by stroke survivors, using mapping provided by Michie et al. 2014 (p113-114). TDF = Theoretical Domains Framework.*

These intervention functions were screened to remove any that obviously did not meet APEASE criteria (affordability, practicability, effectiveness and cost-effectiveness, acceptability, side-effects/safety; equity). The intervention functions ‘coercion’ and ‘restriction’ were not carried to the next mapping stage as they seemed unlikely to be acceptable. Remaining intervention functions were carried to the next mapping stage (Table App2b). It was uncertain whether ‘incentivisation’ would be acceptable, or what the reward might be for this specific behaviour and target (stroke survivor), but this intervention function was not ruled out at this stage.

| **Intervention function** | **Potential policy categories** | **Most used BCTs** |
| --- | --- | --- |
| **Education** | Communication/marketing  Guidelines  Regulation  Legislation  Service provision | Information about social and environmental consequences  Information about health consequences  Feedback on behaviour  Feedback on outcome(s) of the behaviour  Prompts/cues  Self-monitoring of behaviour  *Less commonly used BCTs include:*  Information about emotional consequences  Information about others’ approval |
| **Persuasion** | Communication/marketing  Guidelines  Regulation  Legislation  Service provision | Credible source  Information about social and environmental consequences  Information on health consequences  Feedback on behaviour  Feedback on outcome(s) of the behaviour  *Less commonly used BCTs include:*  Verbal persuasion about capability  Information about emotional consequences  Salience of consequences  Information about others’ approval |
| **Training** | Guidelines  Fiscal measures  Regulation  Legislation  Service provision | Demonstration of the behaviour  Instruction on how to perform a behaviour  Feedback on the behaviour  Feedback on outcome (s) of behaviour  Self-monitoring of behaviour  Behavioural practice/rehearsal  *Less commonly used BCTs include:*  Habit formation  Graded tasks |
| **Environmental restructuring** | Guidelines  Fiscal measures  Regulation  Legislation  Environmental/social planning | Adding objects to the environment  Prompts/cues  Restructuring the physical environment |
| **Modelling** | Communication/marketing  Service provision | Demonstration of the behaviour |
| **Enablement** | Guidelines  Fiscal measures  Regulation  Legislation  Environmental/social planning  Service provision | Social support (unspecified)  Social support (practical)  Goal setting (behaviour)  Goal setting (outcome)  Adding objects to the environment  Problem solving  Action planning  Self-monitoring of behaviour  Restructuring the physical environment  Review behaviour goal(s)  Review outcome goal (s).  *Less commonly used BCTs include:*  Reduce negative emotions  Monitoring of emotional consequences |
| **Incentivisation** | Communication/marketing  Guidelines  Fiscal measures  Regulation  Legislation  Service provision | Feedback on behaviour  Feedback on outcome(s) of behaviour  Monitoring of behaviour by others without feedback  Monitoring of outcome(s) of behaviour without feedback  Self-monitoring of behaviour  *Less commonly used BCTs include:*  Rewarding completion  Social reward  Material reward (behaviour)  Reward (outcome)  Incentive  Behavioural contract  Commitment  Discrepancy between current behaviour and goal |
|  | | |

***Table App2b:*** *Mapping identified intervention functions to potential policy categories and BCTs (Behaviour Change Techniques) for the behaviour of cleaning teeth/mouth by stroke survivors. This mapping is based on the identification and mapping of intervention functions, policy categories and BCTs provided by Michie et al. 2014 (p136-137 & p151-155.*

**APPENDIX 3 - Draft Service Blueprint**

The draft service blueprint was developed to define opportunity areas for the new intervention, relative to the Improved User Journey. The concepts proposed in the *Physical Evidence* row, across the top of the blueprint, were the foundation for the intervention components. The columns represent the notional owner of each step in the journey, and are colour coded to Healthcare (pink), Social Care (orange) or Voluntary Community Sector (blue).

***[A full-size scalable graphic will be available to the Journal]***


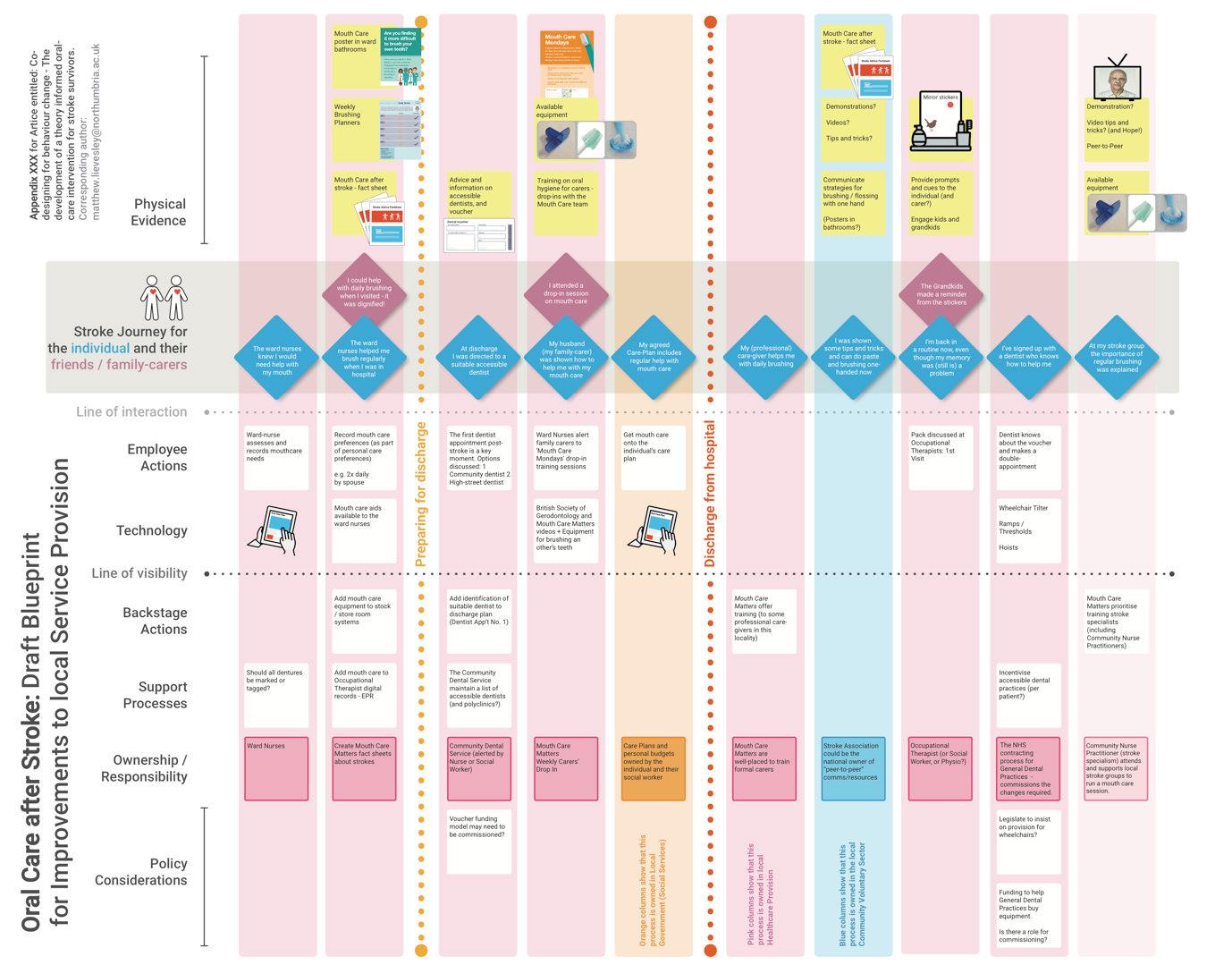


**APPENDIX 4 – Alignment and prioritisation of Intervention Components**

Additional data from the behavioural analysis, illustrating the alignment of appropriate BCTs, Intervention Functions and Policy Categories to each intervention component proposed.

*NOTE: The target behaviours are usually those originally specified: ‘cleaning teeth/mouth’ and ‘accessing dental care’. Additional, intermediate target behaviours (identified during the research process) are indicated where relevant.*

|  | **Intervention Component and modality** | **Description/Content** | **Behaviour Change Techniques (BCTs)** | **Intervention Functions** | **Policy Categories** |
| --- | --- | --- | --- | --- | --- |
| C1 | POSTER PROMPT  for the Ward bathroom | The poster asks “Are you finding it more difficult to brush your own teeth?” to prompt stroke patients to ask health  professionals to check this before discharge from the ward. | Target: stroke survivors  7.1: Prompts/cues | Environmental restructuring | Communication /marketing  Environmental/ social planning |
| C2 | FACTSHEET Mouth care after Stroke.  Given on admission to the stroke ward, at Mouth Care Mondays (C5) and at Stroke Groups (C13) | To help stroke-survivors and carers understand what to do differently for mouth care after stroke.  Contents: brushing – why it’s important and how to do it effectively, brushing someone else’s teeth (with video demos), useful equipment tips, dentures. | Target: stroke survivors and family carers  4.1 Instruction on how to perform the behaviour  5.1 Information about health consequences,  5.3 Information about social/environmental consequences;  6.1 Demonstration of behaviour [if follow link]  [2.2 – feedback on behaviour – advised on how to gain feedback] | Education  Training  Modelling | Communication /marketing |
| C3 | WEEK 1 MOUTH CARE CHART  Bedside chart. Tear-off mini brushing-packs for the first week on the stroke ward. | Wall-fixed, day-by-day chart with tear-off packs. Pack contents – pre-pasted brush, cleansers etc. Personalised to the individual when issued. Stays on view on the ward and tracks when mouth care was last given (as each pack is torn away). | Target: stroke survivors and family carers  2.3 Self-monitoring of behaviour  7.1 Prompts/ cues  12.5 Adding objects to environment; | Environmental restructuring  Enablement | Environmental/  social planning |
| C4 | WEEK 2 MOUTH CARE CHART  Bedside chart. | Wall-fixed, day-by-day chart with pockets for your own brush, paste and equipment. Personalised to the individual when issued. By moving the brush along to the next pocket after brushing it tracks when mouth care was last given. | Target: stroke survivors and family carers  2.3 Self-monitoring of behaviour  7.1 Prompts/ cues  12.5 Adding objects to environment; | Environmental restructuring  Enablement | Environmental/ social planning |
| C5 | MOUTH CARE MONDAYS  Carers’ weekly drop-in – on the ward. One-hour draft agenda (and poster C6) | A once-a-week drop-in for family carers to have mouth care techniques such as brushing someone else’s teeth and see equipment demonstrated. This could include practicing the technique with support from the trainer.  FACTSHEET (C2) given to participants. | Target: family carers  3.1 Social support (unspecified)  4.1 Instruction on how to perform the behaviour;  5.1 Information about health consequences,  5.3 Information about social/environmental consequences;  6.1 Demonstration of the behaviour  8.1 Behavioural practice [if feasible]  12.5 Adding objects to the environment [if feasible to provide useful equipment] | Education  Training  Modelling | Service provision |
| C6 | MOUTH CARE MONDAYS  Poster for the ward’s notice-board and/or Family Room | Poster promoting the next and nearest Mouth Care Mondays session on that site. Also raises awareness of the need for good mouth care. | Target: family carers. Target behaviour: attending Mouth Care Mondays.  4.1 Instruction on how to perform the behaviour  7.1 Prompts/cues | Environmental restructuring | Communication/marketing  Environmental/social planning |
| C7 | EPR CHANGE 1  (software fix)  For action by Occupational Therapists | A specific check for independent oral self-care, added to the OT’s electronic checklist, alongside existing checks, such as walking, toileting and climbing stairs. | Target: Occupational Therapist  Target behaviour: check for independent oral self-care  2.3 Self-monitoring of behaviour  7.1 Prompts/cues | Environmental restructuring  Enablement | Service provision |
| C8 | DENTAL VOUCHER  Issued to the named recipient at discharge. It secures a *double* dental appointment for the named recipient, ensuring they aren’t rushed. | The voucher guides a staff-led discharge discussion about the stroke survivor’s access to an appropriate dentist.  A QR-code links to an index of NHS accessible dentists by postcode.  A voucher format has urgency, so it encourages display at home to prompt action.  The Dentist retains the voucher to claim back two units-of-activity (UOAs) for the longer appointment.  A second QR-code, for Dentists, links to stroke-specific gerodontology guidance. | Target: stroke survivors  7.1 Prompts/cues  Target: dental practice  10.1 Material incentive (behaviour)  10.2 Material reward (behaviour) | Environmental restructuring  Enablement | Service provision  Fiscal measures  Regulation |
| C9 | EPR CHANGE 2  (software fix)  For action by Hospital-based Social Workers | A specific check for independent oral self-care, added to the SW’s electronic Care Needs Assessment checklist, alongside other checks on everyday-living. | Target: Social Worker. Target behaviour: check for independent oral self-care  2.3 Self-monitoring of behaviour  7.1 Prompts/cues | Environmental restructuring  Enablement | Service provision |
| C10 | TRAINING ON MOUTH CARE – FOR PROFESSIONAL CAREGIVERS | No proposal about delivery format was made, as another specific project on this topic was ongoing at the time. | Target: professional caregivers  Likely to include:  4.1 Instruction on how to perform the behaviour;  5.1 Information about health consequences,  5.3 information about social/environmental consequences;  6.1 Demonstration of the behaviour  8.1 Behavioural practice | Education  Training | Service provision |
| C11 | PEER-TO-PEER FILMS  Hopeful online messages, shared by stroke-survivors | With potential to be hosted within a range of existing online fora. Three short films by stroke-survivors, on recovery and successfully adapting to life-after-stroke. | Target: stroke survivors  Likely BCTs (depending on final content of films):  9.1 Credible source  15.1 Verbal persuasion about capability  16.3 Vicarious consequences  6.1 Demonstration of the behaviour | Education  Persuasion  Modelling | Communication/marketing |
| C12 | MEMORY STICKERS  For the home environment – given at discharge | For the families of stroke-survivors to compose *very-personal* reminders, in place, at home. E.g. a grandchild uses the pack to create a message/image for a bathroom mirror. | Target: stroke survivors  7.1 – Prompts/cues | Environmental restructuring | Communication/marketing  Environmental/ social planning |
| C13 | EXPERT INPUT AT STROKE GROUPS  Once or twice a year | Mouth Care Q+A and equipment demos at local Stroke Groups.  Agenda based on (C5) Mouth Care Mondays and session led by either Dental Therapists or SLTs.  FACTSHEET (C2) given to participants. | Targets: stroke survivors and family carers  3.1 Social support (unspecified)  4.1 Instruction on how to perform the behaviour  5.1 Information about health consequences,  5.3 Information about social/environmental consequences;  6.1 Demonstration of the behaviour  8.1 Behavioural practice [if feasible]  12.5 Adding objects to the environment [if feasible to provide useful equipment] | Education  Training  Modelling | Service provision |
|  | | | | | |

Four of these original thirteen proposed components were not developed beyond Workshop 3. *Peer-to-peer films* (C11) were not prioritised by workshop participants. Changes to checklists in the hospital *EPR system* (C7, C9) were forecast to take more than two-years to implement. So, instead, tangible prompts C1 and C8 were developed, to prompt timely conversations to flag issues, even in the absence of a software-mandated check. *Training on mouth care for formal care-givers* (C10) was already being addressed by a separate project.
